# GSK3β phosphorylated tau filaments are probably not like those from AD brains

**DOI:** 10.1073/pnas.2503730122

**Published:** 2025-07-07

**Authors:** Sjors H. W. Scheres

**Affiliations:** ^a^Medical Research Council, Structural Studies Division, Laboratory of Molecular Biology, Cambridge CB2 0QH, United Kingdom

The filamentous assembly of tau is a hallmark of multiple neurodegenerative diseases, including Alzheimer’s disease (AD). In 2017, we introduced software for helical reconstruction of electron cryomicroscopy (cryo-EM) images (1), and we used this software to determine the structures of tau paired helical and straight filaments (PHFs and SFs) extracted from the brain of an individual with AD (2). Since then, we and others have used this software to determine more than five hundred amyloid structures (3). These combined efforts have shown that a given protein can adopt multiple amyloid structures and that, for tau and other proteins, specific folds characterize different diseases (4).

**Fig. 1.**
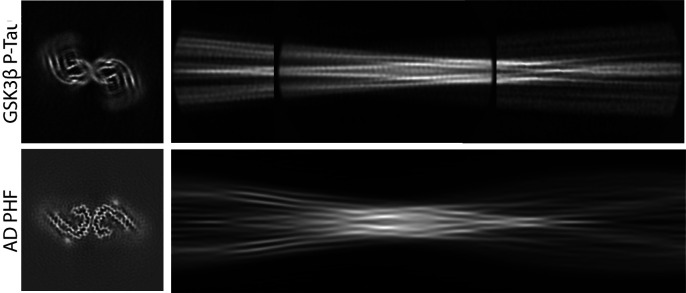
(*A*) Cross-section of the reconstruction of GSK3β phosphorylated tau filaments. (*B*) Cross-section of the reconstruction of an AD PHF, filtered at 5 Å. (*C*) Assembly of three 2D class averages, comprising part of the cross-over of GSK3β phosphorylated tau filaments. (*D*) Projection of the cross-over for the AD PHF shown in *B*. The map in panel *A* and particle images used to calculate the 2D class averages in panel *B* were provided by the authors upon request. All panels are on the same scale.

Recently, Chakraborty et al. reported that phosphorylation with the serine/threonine kinase GSK3β catalyzes the assembly of recombinant tau (5). They used our software to calculate a cryo-EM reconstruction of the resulting filaments. The authors posit that our software overestimated the resolution (3.8 Å), and claim that the correct resolution is ~5 Å. However, rather than suffering from overestimated resolution, the reconstruction most likely represents an incorrect local minimum of the helical reconstruction process. This well-documented problem (6, 7) is the reason why users of our software are advised to remain critical of unexpected features in the reconstruction (8). The reconstruction of the GSK3β phosphorylated tau filaments shows many unexpected features: Densities are disconnected and without recognizable protein-like features (Fig. 1*A*). For comparison, a reconstruction of an AD PHF, at 5 Å resolution, shows fully connected amino acid main chains, with side chain densities depending on their size and the resolution (Fig. 1*B*).

We published detailed protocols on how to deal with local minima in amyloid structure determination (7, 8). These instructions were not followed for the GSK3β phosphorylated tau filaments. Because the resulting reconstruction is likely incorrect, it cannot distinguish between the many different structures that have been reported for tau filaments to date. Comparison of a projected cross-over of an AD PHF (Fig. 1*D*) and an assembly of reference-free 2D class averages of GSK3β phosphorylated tau filaments (Fig. 1*C*) suggests that they are different. Therefore, the conclusions that these filaments are “AD-like,” and that they are “more similar to” filaments from extracellular vesicles than from brain extracts, are not justified by the data presented by Chakraborty et al. Cryo-EM structure determination, when done correctly, leaves no ambiguity as to whether two structures are the same or not, and incorrect structures should not become part of the scientific discourse. Submission of cryo-EM reconstructions to the Electron Microscopy Data Base (EMDB) (9), and of unprocessed images to EMPIAR (10), allows scrutiny by the scientific community; the former should be mandated by the journal.
